# Supplementary material for: Members of Venezuelan Equine Encephalitis complex entry into host cells by clathrin-mediated endocytosis in a pH-dependent manner
Source: Sci Rep. 2022 Aug 25;12:14556. doi: 10.1038/s41598-022-18846-w (PMC9411563; doi:10.1038/s41598-022-18846-w)
Supplement: Supplementary file 1 — Supplementary Information. [file 41598_2022_18846_MOESM1_ESM.pdf]

Members of Venezuelan Equine Encephalitis Complex entry into host cells by clathrin-mediated endocytosis in a pH-dependent manner

Lucia Maria Ghietto[1, †], Pedro Ignacio Gil[1, †], Paloma Olmos Quinteros[1], Emiliano Gomez[1], Franco Martin Piris[1], Patricia Kunda[1, 3], Marta Contigiani[1], Maria Gabriela Paglini[1, 2, *].

[1]Instituto de Virología "Dr. JM Vanella", Facultad de Ciencias Médicas, Universidad Nacional de Córdoba, Córdoba, Argentina.

[2]Instituto de Investigación Médica Mercedes y Martín Ferreyra, INIMEC-CONICET-Universidad Nacional de Córdoba, Córdoba, Argentina.

[3]Centro de Investigación en Medicina Traslacional "Severo Amuchástegui" (CIMETSA), Instituto Universitario Ciencias Biomédicas Córdoba (IUCBC), Naciones Unidas 420, Córdoba, Argentina

[†]These authors have contributed equally to this work

*** Corresponding author:**
María Gabriela Paglini, Ph.D.
Instituto de Virología "Dr. JM Vanella"
Facultad de Ciencias Médicas,
Universidad Nacional de Córdoba, Córdoba, Argentina.
Phone: +54-351-4681465 (ext. 105) Fax: +54-351-4695163.
E-mail: gpaglini@immf.uncor.edu

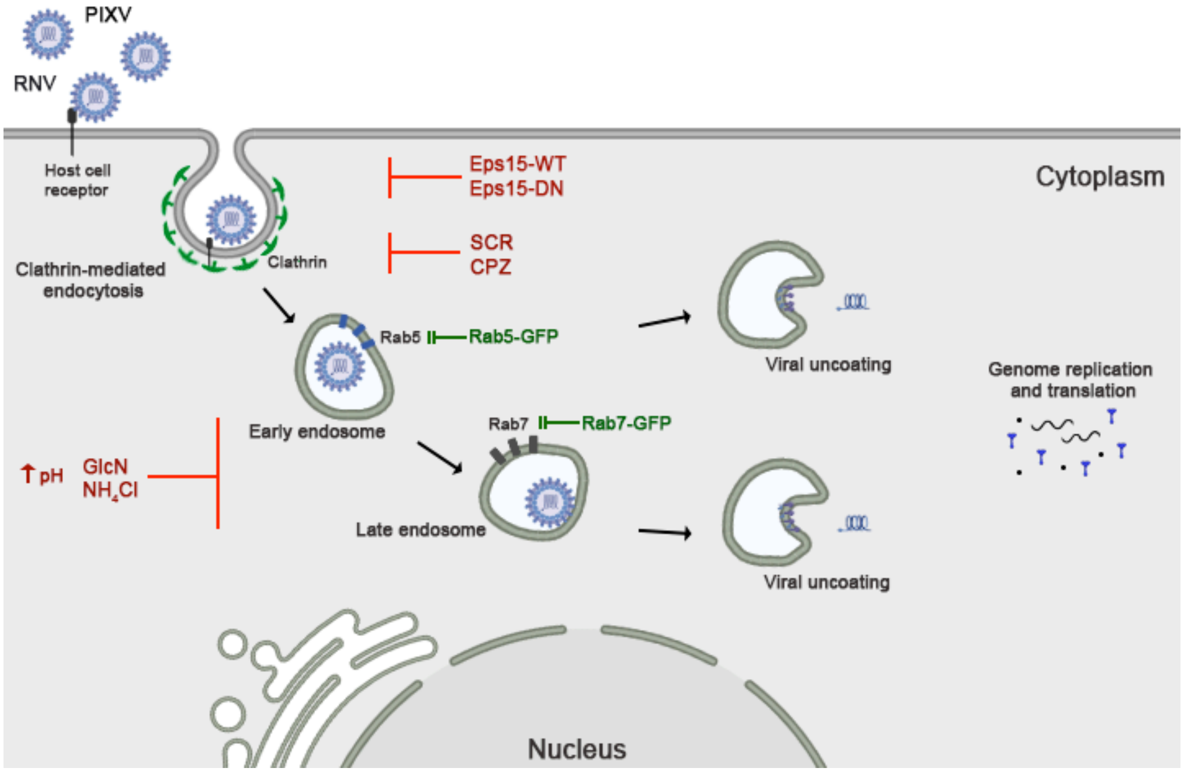

Supplementary Figure S1: **Pixuna and Rio Negro virus, members of Venezuelan Equine Encephalitis Complex, entry into host cells by clathrin-mediated endocytosis in a pH-dependent manner. PIXV**: Pixuna virus. **RNV**: Rio Negro virus. **Eps15-WT**: Eps15 wild-type construct. **Eps15-DN**: dominant negative DN Δ95/295 mutant construct. **SCR**: Sucrose. **CPZ**: Chlorpromazine. **Rab5-GFP**: early endosomal marker. **Rab7-GFP**: late endosomal marker. **GlcN**: Glucosamine. **NH_4Cl**: ammonium chloride.
